# Human restricted *CHRFAM7A* mediates neuronal and innate immune gain of function

**DOI:** 10.1002/alz.084418

**Published:** 2025-01-09

**Authors:** Kinga Szigeti, Ivanna Ihnatovych, Ryu Dorn, Nicolas Rosas, Eduardo Cortes Gomez, Gregory Wilding, Wang Jianmin

**Affiliations:** ^1^ University at Buffalo, Buffalo, NY USA; ^2^ Roswell Park Cancer Institute, Buffalo, NY USA

## Abstract

**Background:**

Understanding the fundamental differences between the human and pre‐human brain is a prerequisite for designing meaningful models and therapies for AD. Expressed *CHRFAM7A*, a human restricted gene with carrier frequency of 75% in the human population predicts profound translational significance.

**Method:**

The physiological role of CHRFAM7A in human brain is explored using multiomics approach on 600 post mortem human brain tissue samples (ROSMAP). The emerging pathways are tested and validated in an isogenic hiPSC model of *CHRFAM7A* knock‐in neurons and monocytes. In vivo validation is performed in a double blind pharmacogenetic study on the effect of nicotinic acetyl‐choline esterase inhibitor therapy. The effect of *CHRFAM7A* carrier status was assessed in two paradigms: response to drug initiation and disease modifying therapy (DMT) effect over an 8 year observation period. Fitted general linear model independent variables included age, sex, and medication regimen at the time of the first MMSE, APOE4 carrier status (0, 1 or 2 alleles as categorical variables) and CHRFAM7A genotype.

**Result:**

*CHRFAM7A* is identified as a modulator of intracellular calcium dynamics and an upstream regulator of Rac1. Rac1 activation re‐designs the actin cytoskeleton leading to dynamic actin driven remodeling of membrane protrusion and a switch from filopodia to lamellipodia. Phenotypic readouts in both the neuronal and monocytic lineages confirm actin cytoskeleton gain of function. The actin cytoskeleton reorganization shifts dendritic spine differentiation from filopodia towards spines with increased head area to stem diameter ratio resulting in increased synapse clustering (“high quality wellcro”) in neurons. In monocytes, the reorganized actin cytoskeleton leads to increased invasion, phagocytosis and cytokine release. These data suggest a shift from neurodegenerative to neuroinflammatory mechanism in 75% of AD patients. From the cholinergic treatment perspective incorporation into the a7 nAChR pentamer results in a hypomorphic receptor, which has diminished response to pharmacological modulation. Proof of principle pharmacogenetic study found a CHRFAM7A effect on treatment response both in the acute response and DMT paradigms.

**Conclusion:**

Human restricted *CHRFAM7A* defines the biological background for AD pathology by shifting neurodegeneration to neuroinflammation in a 25‐75% split. Introducing a *CHRFAM7A* model into the target selection workflow may increase clinical trial success.

